# Recombinant Pichinde viral vector expressing tuberculosis antigens elicits strong T cell responses and protection in mice

**DOI:** 10.3389/fimmu.2023.1127515

**Published:** 2023-02-08

**Authors:** Natalie M. Kirk, Qinfeng Huang, Sophia Vrba, Mizanur Rahman, Alisha M. Block, Hannah Murphy, Dylan W. White, Sarah B. Namugenyi, Hinh Ly, Anna D. Tischler, Yuying Liang

**Affiliations:** ^1^ Department of Veterinary and Biomedical Sciences, College of Veterinary Medicine, University of Minnesota, St. Paul, MN, United States; ^2^ Department of Microbiology and Immunology, School of Medicine, University of Minnesota, Minneapolis, MN, United States

**Keywords:** viral vector based vaccines, tuberculosis vaccine, Pichinde virus vector, vaccine immunity, T cell vaccines, T cell immunity, Mtb mouse model

## Abstract

**Introduction:**

Tuberculosis (TB) caused by *Mycobacterium tuberculosis (Mtb)* remains a major global health threat. The only available vaccine Bacille Calmette-Guérin (BCG) does not prevent adult pulmonary TB. New effective TB vaccines should aim to stimulate robust T cell responses in the lung mucosa to achieve high protective efficacy. We have previously developed a novel viral vaccine vector based on recombinant Pichinde virus (PICV), a non-pathogenic arenavirus with low seroprevalence in humans, and have demonstrated its efficacy to induce strong vaccine immunity with undetectable anti-vector neutralization activity.

**Methods:**

Using this tri-segmented PICV vector (rP18tri), we have generated viral vectored TB vaccines (TBvac-1, TBvac-2, and TBvac-10) encoding several known TB immunogens (Ag85B, EsxH, and ESAT-6/EsxA). A P2A linker sequence was used to allow for the expression of two proteins from one open-reading-frame (ORF) on the viral RNA segments. The immunogenicity of TBvac-2 and TBvac-10 and the protective efficacy of TBvac-1 and TBvac-2 were evaluated in mice.

**Results:**

Both viral vectored vaccines elicited strong antigen-specific CD4 and CD8 T cells through intramuscular (IM) and intranasal (IN) routes as evaluated by MHC-I and MHC-II tetramer analyses, respectively. The IN inoculation route helped to elicit strong lung T cell responses. The vaccine-induced antigen-specific CD4 T cells are functional, expressing multiple cytokines as detected by intracellular cytokine staining. Finally, immunization with TBvac-1 or TBvac-2, both expressing the same trivalent antigens (Ag85B, EsxH, ESAT6/EsxA), reduced *Mtb* lung tissue burden and dissemination in an aerosol challenge mouse model.

**Conclusions:**

The novel PICV vector-based TB vaccine candidates can express more than two antigens *via* the use of P2A linker sequence and elicit strong systemic and lung T cell immunity with protective efficacy. Our study suggests the PICV vector as an attractive vaccine platform for the development of new and effective TB vaccine candidates.

## 1 Introduction

Tuberculosis (TB) is a predominantly pulmonary disease caused by *Mycobacterium tuberculosis* (*Mtb*), with more than 10 million new cases of active disease and 1.6 million deaths attributed to the infection in 2021 (1). *Mtb* is also estimated to cause asymptomatic latent infections in one quarter of the world’s population (2), each of whom has a 5-10% lifetime risk of developing active TB disease (3). The only licensed TB vaccine, Bacille Calmette-Guérin (BCG), is effective at preventing severe childhood meningeal and miliary TB, but does not prevent adult pulmonary TB (4, 5). With multidrug-resistant (MDR) and extensively drug-resistant (XDR) *Mtb* strains becoming more common, development of new and effective TB vaccines is a top global health priority (1). This need was highlighted in 2020 during the COVID-19 pandemic, which caused disruptions in disease detection and reporting that led to increased TB deaths (3). TB vaccine development has accelerated in recent years with several whole cell-derived and subunit vaccines in current or recently completed clinical trials. A recent phase 2b clinical trial of the recombinant fusion protein vaccine candidate (M72/AS01_E_) in latently infected adults elicited immune responses and provided 54.0% protection from active TB disease over a 3-year study period (6). These promising clinical trial results indicate that an effective TB vaccine is likely achievable. An expanded pipeline of diverse TB vaccine candidates is needed to identify the vaccine modalities and antigens that confer robust immune protection.

Control of *Mtb* infection is generally believed to require cellular immune responses (7, 8). CD4 Th1 cells can activate phagocytes by producing the IFNγ and TNFα cytokines and cytotoxic CD8 T cells can kill *Mtb*-infected cells (9). In TB vaccine-immunized non-human primates (NHPs), T cell subsets associated with protection include multifunctional antigen-specific Th1/Th17 CD4 and CD8 T cells (10, 11), and *Mtb*-specific CD4 and CD8 T cells with circulating central memory or tissue-resident memory phenotypes (12). Therefore, new TB vaccines should aim to stimulate robust cellular immunity in the lung mucosa to achieve high protective efficacy.

Viral vector vaccines mimic natural infection and elicit robust cell-mediated and humoral immune responses without the need of adjuvant (13). Many viral vectored vaccines have been approved for veterinary diseases (13), and several were approved for human usage in recent years. A Zaire ebolavirus vaccine based on a recombinant vesicular stomatitis virus vector (ERVEBO^®^) was approved by the Food and Drug Administration (FDA) in 2019 (14). COVID-19 vaccines based on adenovirus vectors, such as the adenovirus type 26 (Ad26)-based Janssen vaccine and the Chimpanzee adenovirus vector (ChAd)-based Oxford/AstraZeneca vaccine, have been used during the pandemic under emergency use authorizations from the FDA (15) or the World Health Organization (WHO) (16, 17). Viral vectors have also been explored for TB vaccine development. Heterologous prime-boost of antigens delivered by ChAd and modified vaccinia virus Ankara (MVA) vectors induced *Mtb*-specific immune responses in the lung but not protection in NHPs (18). A rhesus CMV vector multivalent vaccine (RhCMV/TB) reduced *Mtb* infection and disease in NHPs (12), but the strict species specificity of CMVs prevents evaluation of RhCMV in other animal models and in humans. Phase 1 clinical trials have shown that two viral vectors, human adenovirus 5 (hAd5) (19) and MVA (20), are safe and elicit both systemic and mucosal immunity in trial participants. These studies set the stage for further development of mucosal vaccines targeting the respiratory system.

We have developed a novel viral vaccine vector based on a tri-segmented recombinant Pichinde virus (PICV) rP18tri, which can accommodate two additional open-reading frames (ORFs) (21, 22). PICV is an arenavirus first isolated from rice rats in Colombia and is not known to cause human disease (23). The rP18tri vector is attenuated both *in vitro* and *in vivo* and causes a self-limiting infection in animals without any adverse effects or shedding of detectable virus in blood or body fluids (21). For these reasons, rP18tri has an increased safety profile compared to other live viral vectors. The low seroprevalence of PICV in human populations (23) also mitigates concern over pre-existing anti-vector immunity that is known for some widely used viral vectors, including hAd5 (24). The rP18tri vector induces even stronger immune responses upon a homologous boost and can be repeatedly administered without significantly decreasing the vaccine immunity (21). The live-attenuated rP18tri vector elicits strong adaptive immunity (21), possibly because PICV targets antigen-presenting cells early in the infection, which enhances antigen presentation and increases the frequency and avidity of specific adaptive immunity. rP18tri can be given to many mammalian host species through various routes such as intramuscular (IM), intraperitoneal (IP), intranasal (IN), and oral (21). These features make rP18tri an attractive vector for developing strong mucosal immunity in various host species.

Here, we provide a proof-of-concept that rP18tri-based multivalent TB vaccine candidates can stimulate robust systemic and lung T cell immune responses with protective efficacy in mice. We generated three viral vectored vaccines TBvac-1, TBvac-2, and TBvac-10 expressing multiple *Mtb* antigens including Ag85B, EsxH, and ESAT-6/EsxA. We characterized the quantity, phenotype, and functionality of T cells elicited by these viral vectored vaccines and evaluated protection in a virulent *Mtb* challenge mouse model. Our study suggests the PICV vector as an attractive vaccine platform for development of new effective TB vaccine candidates.

## 2 Materials and methods

### 2.1 Ethics statement

Research conducted for this manuscript was approved by the Institutional Biosafety Committee at the University of Minnesota, Twin Cities, under the protocol ID 2008-38359H and 2010-38519H. All animal procedures were approved by the Institutional Animal Care and Use Committee (IACUC) of University of Minnesota, Twin Cities, under the protocol ID 2006-38175A and 2005-38161A.

### 2.2 Mammalian cells, viruses, and *Mtb* strain

BHK21 Baby hamster kidney cells and Vero African green monkey kidney cells were grown in DMEM media (Fisher Scientific) with 10% fetal bovine serum (FBS) (Sigma) and 50 μg per ml penicillin and streptomycin (Invitrogen-LifeTechnologies). BSRT7-5 cells, obtained from K.K. Conzelmann (Ludwig-Maximilians-Universität, Germany), are BHK-21 cells stably expressing T7 RNA polymerase. BSRT7-5 cells were grown in minimal essential medium (MEM) (Invitrogen-LifeTechnologies) with 10% FBS, 1 μg/ml Geneticin (Invitrogen-LifeTechnologies), and 50 μg/ml penicillin-streptomycin. Recombinant PICV-vectored TB vaccines were amplified in BHK-21 cells. Infectious virus titer was determined by viral plaque assay in Vero cells as described previously (25). *Mycobacterium tuberculosis* Erdman is a fully virulent isolate and a BSL-3 agent. *Mtb* was routinely cultured at 37˚C with aeration in Middlebrook 7H9 liquid medium (Difco), which was supplemented with 10% albumin-dextrose-saline (ADS), 0.5% glycerol and 0.1% Tween-80.

### 2.3 Proteins, peptides, tetramers, and antibodies

*Mtb* proteins, peptide arrays, and antibodies were obtained from BEI Resources, NIAID, NIH. These include purified Ag85B (NR-14857) and ESAT-6/EsxA (NR-49424) proteins from *Mtb* strain H37Rv, peptide arrays of *Mtb* Ag85B (NR-34828) and ESAT-6 (NR-50711), rabbit polyclonal anti-ESAT6 (NR-13803) and anti-Ag85 Complex (NR-13800) antisera. Phycoerythrin (PE)-conjugated EsxH MHC-I tetramer (H-2K(b) IMYNYPAM), PE-conjugated MHC-II control and Ag85B tetramers were obtained from the NIH Tetramer Core Facility at Emory University. Antibodies used for T cell analysis of immunized mice were purchased from commercial sources, including allophycocyanin (APC)-labeled anti-CD3 (17A2) (Biolegend), fluorescein isothiocyanate (FITC)-labeled anti-CD4 (Biolegend), peridinin chlorophyll protein (PerCP)-Cy5.5-labeled anti-CD8 (53-6.7) (eBioscience), brilliant violent (BV) 510-labelled anti-CD44 (Biolegend), APC-Cy7 anti-CD69 (Biolegend), and BV421 anti-CD103 (BD Biosciences).

### 2.4 Generation of rP18tri-based TB vaccines

**Figure 1:**
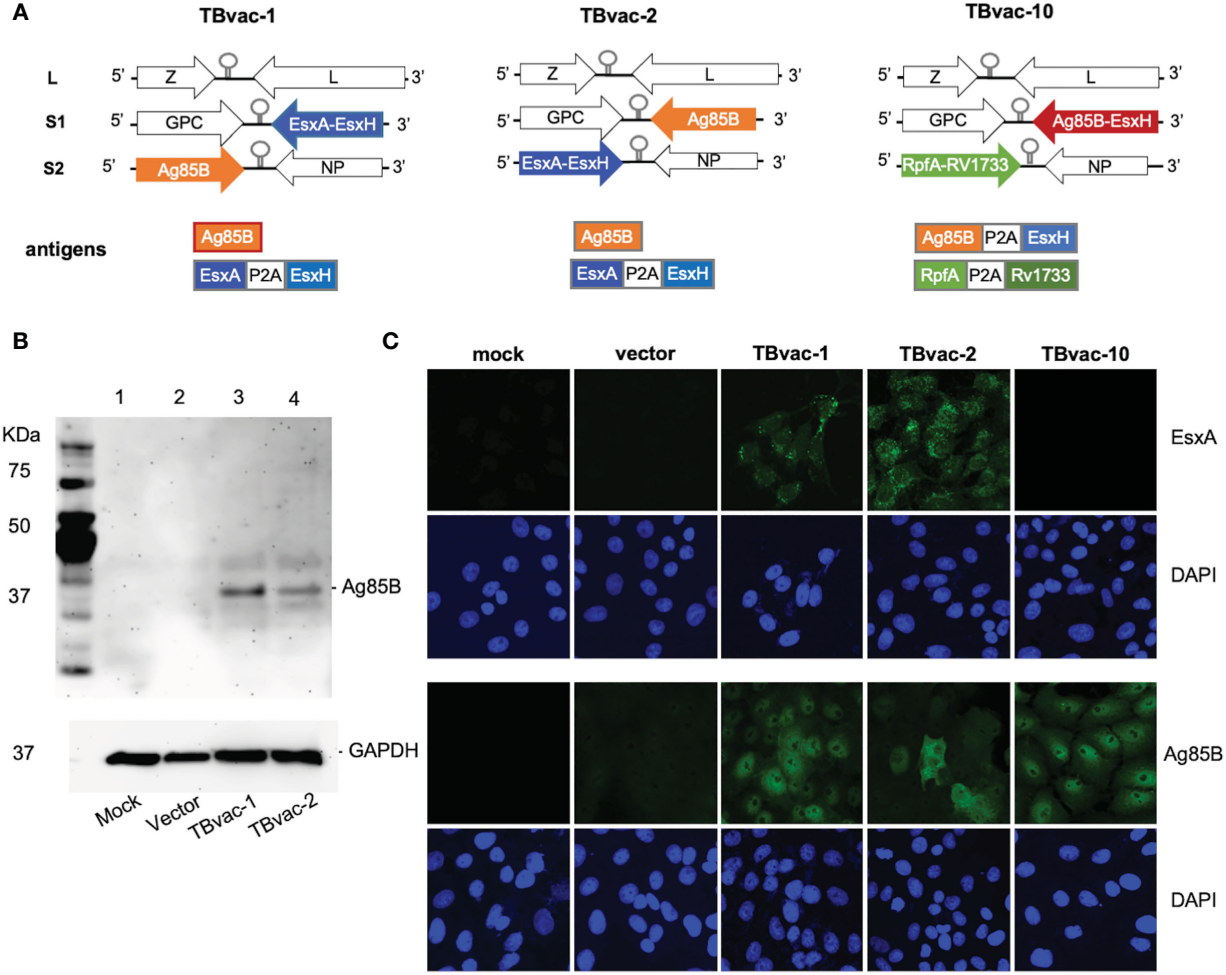
Generation of rP18tri-based viral vector vaccines encoding *Mtb* antigens. **(A)** Genomic organization of the viral vectored TB vaccines (TBvac-1, TBvac-2 and TBvac-10), with the inserted *Mtb* antigens shown below. The P2A linker sequence was used to express two antigens from one ORF. **(B)** Detection of *Mtb* Ag85B antigen expression by Western blotting. Vero cell lysates collected 24 hours after mock infection or infection with vector, TBvac-1, or TBvac-2 were analyzed by Western blotting with anti-Ag85B and anti-GAPDH antibodies. **(C)** Detection of *Mtb* antigen expression in the infected Vero cells by IFA with anti-ESAT-6 and anti-Ag85B antibodies.

Recombinant tri-segmented PICV (rP18tri)-based TB vaccines were constructed as described previously (21, 22). Sequences of *Mtb* antigens Ag85B (NP_216402), EsxH/TB10.4 (NP_214802), ESAT6/EsxA (YP_178023), Rv1733c (NP_216249), and RpfA (NP_215382) were obtained from GenBank. Gene fragments encoding Ag85B single gene and dual antigens (EsxA-EsxH, Ag85B-EsxH, and RpfA-Rv1733c) linked by a P2A linker sequence (GSGATNFSLLKQAGDVEENPGP) (26) were codon optimized for human cell expression and chemically synthesized by Genewiz (Azenta Life Sciences). These gene fragments were cloned into the S1 or S2 vector of the rP18tri reverse genetics system between Kpn I and Xho I sites (21, 22). The resulting plasmids were verified by restriction enzyme digestion and sequencing. To rescue recombinant rP18tri vector-based TB vaccines, BSRT7-5 cells seeded at 1.5x10^5^ cells per ml in a 6-well plate were transfected with three plasmids expressing the L segment, the S1 and S2 segments encoding respective antigens as illustrated in **Figure 1A**, using Lipofectamine™ 3000 Transfection Reagent (Thermo Fisher Scientific, L3000015). From 2 to 4 days post-transfection, the supernatants were collected for plaque assay on Vero cells. Individual plaques were picked to infect BHK-21 cells on 10-cm plates for 48 h to prepare viral stocks. Viral RNA was extracted from viral stocks using the QIAamp Viral RNA kit (Qiagen, USA), subjected to RT-PCR and sequencing to confirm the identity of viral vectored TB vaccines. TBvac-1 encodes EsxA-EsxH on the S1 segment and Ag85B on the S2 segment, while TBvac-2 encodes Ag85B on the S1 segment and EsxA-EsxH on the S2 segment. TBvac-10 encodes Ag85B-EsxH on the S1 segment and RpfA-Rv1733c on the S2 segment.

### 2.5 Plaque assay to quantify viral titer of rP18tri-based TB vaccines

Vero cells in six-well plates were washed and infected with 0.5 ml of viruses in 10-fold serial dilutions. After incubation for 1 h at 37°C, the infection medium was removed. Fresh MEM supplemented with 0.5% agar and 10% FBS were added to the cells, which were cultured for 4 days at 37°C. Plaques were stained overnight with neutral red solution diluted (1:50) in 0.5% agar–MEM–10% FBS.

### 2.6 Detection of antigen expression in TBvac-infected cells

Vero cells grown on coverslips were mock infected or infected with the rP18tri vectors alone or respective TBvac viruses at MOI of 0.1. At 24 hours post infection (hpi), cells were fixed with 4% paraformaldehyde for 15 min at room temperature, and washed three times with phosphate buffer saline (PBS) (Invitrogen-LifeTechnologies). Cells were treated with 0.1% Triton X-100 for 12 min followed by incubation with rabbit polyclonal antibody against Ag85B or ESAT6 (BEI) for 1 h. After washing, cells were incubated with goat anti-rabbit Alexa Fluor-488 (Invitrogen) for 1 hr at room temperature. The cells were washed three times and mounted on a glass slide for examination by confocal microscopy.

### 2.7 Mouse immunization and tissue collection

For immunogenicity studies, female six- to eight-week-old C57BL/6 mice were obtained from Jackson Laboratories and housed for at least one week for acclimatization. Groups of three to five mice were immunized intranasally (IN) or intramuscularly (IM) depending on the study protocol with 1x10^5^ PFU of TBvac-2, TBvac-10, rP18tri vector, or phosphate buffered saline (PBS) as a mock control. To evaluate immune responses after primary immunization, blood was collected into lithium heparin tubes (Greiner Bio-One) *via* the facial vein. Peripheral blood mononuclear cells (PBMCs) were isolated by first lysing red blood cells (RBC) using 1X RBC lysis buffer (eBiosciences) and then washing in PBS with 2% fetal bovine serum (FBS) (Sigma Aldrich). Three weeks after primary immunization, mice were boosted IN or IM with the same vaccine candidate. Seven days after the boost, mice were euthanized by CO_2_ inhalation and the spleen and lung collected into sterile PBS. To isolate splenocytes, spleens were gently crushed through a 40 µm cell strainer and washed with RPMI-1640 medium with L-glutamine (Cytiva HyClone). Red blood cells were lysed using 1X RBC lysis buffer and splenocytes resuspended in PBS with 2% FBS. To isolate lung lymphocytes, lungs were cut into small pieces and incubated for 1 hour at 37C in PBS+5% FBS with 1.5 mg/ml type I collagenase (Worthington Biochemical) and 2 μg/ml DNase I (New England BioLabs). Then, lungs were gently crushed through a 40 µm cell strainer, washed with RPMI-1640/1% FBS and 10mM HEPES (ThermoFisher). Cells were resuspended in 44% Percoll (GE Healthcare), underlain with 67% Percoll, centrifuged, and the lymphocyte layer collected. Lymphocytes were resuspended in PBS with 2% FBS.

### 2.8 IFNγ-based ELISpot assay

ELISpot was performed using Mouse IFNγ ELISpot Development Module (R&D Systems, SEL485) following the manufacturer’s instruction. Briefly, 96-well PVDF-bottom Immunospot plates were coated with anti-mouse IFNγ capture antibody overnight at 4°C. The next day, plates were washed and blocked in 1% bovine serum albumin (BSA)/5% sucrose/PBS. 1x10^5^ splenocytes were added per well and incubated with medium only or with peptide pools of Ag85B or ESAT6/EsxA (2 µg/ml) (BEI) for 18 hours at 37°C and 5% CO2. After incubation, biotinylated anti-mouse IFNγ detection antibody was added and incubated overnight at 4°C. The following day, streptavidin-alkaline phosphatase and BCIP/NBT were added for color development. Spots were recorded using CTL ImmunoSpot Reader and quantified using immunospot Fluox suite software.

### 2.9 Evaluation of antigen-specific CD8 T cells by MHC-I tetramer analysis

The PE-labeled MHC-I tetramer H-2K(b)/EsxH epitope IMYNYPAM was provided by the NIH Tetramer core facility at Emory University (Atlanta, GA). Isolated PBMCs, splenocytes, and lung lymphocytes were incubated with PE-labeled EsxH/H-2K(b) MHC-I tetramer, fixable viability stain 780 (BD Biosciences), APC-labeled anti-CD3 (Biolegend), PerCP-Cy5.5-labeled anti-CD8 (eBioscience), FITC-labeled anti-CD4 (Biolegend), and BV510-labeled anti-CD44 (Biolegend) for one hour at room temperature. Cells were washed three times with PBS/2% PBS. Sample acquisition was performed on FACSCelesta and data analyzed using FlowJo.

### 2.10 Evaluation of antigen-specific CD4 T cells by MHC-II tetramer analysis

The PE-labeled MHC-II Ag85B tetramer I-A(b)/FQDAYNAAGGHNAVF and control tetramer I-A(b)/PVSKMRMATPLLMQA were provided by the NIH Tetramer core facility at Emory University (Atlanta, GA). Isolated PBMCs, splenocytes, and lung lymphocytes were incubated with fixable viability stain 780 (BD Biosciences), APC-labeled anti-CD3 (Biolegend), PerCP-Cy5.5-labeled anti-CD8 (eBioscience), FITC-labeled anti-CD4 (Biolegend), and BV510-labeled anti-CD44 (Biolegend), together with either control or Ag85B PE-labeled MHC-II tetramer. To detect tissue-resident CD4 T cells in the lung, lung lymphocytes were incubated with the same cocktails, together with APC-Cy7-labeled anti-CD69 (Biolegend). Cells were washed three times with PBS/2% PBS. Sample acquisition was performed on FACSCelesta and data analyzed using FlowJo.

### 2.11 Intracellular cytokine staining

Isolated cells were seeded in leukocyte medium (complete RPMI) at 1x10^6^ cells per well and incubated with medium alone or with purified Ag85B/ESAT-6 proteins (each 10 µg/ml) obtained from BEI for 2 hours at 37°C. Cells were further incubated for six hours at 37°C with anti-CD28 antibody and GolgiStop (BD Biosciences). Cells were collected, washed with PBS/2% FBS, and stained with fixable viability dye 780, PerCP-Cy5.5-labeled anti-CD3, FITC-labeled anti-CD8, APC-Cy7-labeled anti-CD4, and PE-Cy7-labeled anti-CD44 for 45 minutes on ice. Cells were then fixed and permeabilized using the Cytofix/Cytoperm kit (BD Biosciences) and stained with BV421-labeled anti-TNFα, PE-labeled anti-IL-2, and APC-labeled anti-IFNγ for 40 minutes on ice. Sample acquisition was performed on FACSCelesta and data analyzed using FlowJo.

### 2.12 *Mtb* mouse aerosol challenge model

Female six to eight-week-old C56BL/6 mice were immunized IM with 1x10^5^ PFU of rP18tri vector, TBvac-1, or TBvac-2 in a prime-boost strategy with a 21-day interval. At 28 days after immunization, vaccinated mice were challenged with ~400 CFU of virulent *Mtb* Erdman, using a Glas-Col Inhalation Exposure System, in a biosafety level 3 (BSL3) laboratory, as previously described (27). A group of unvaccinated mice (n=4) were euthanized at 24 hr post-infection to determine the *Mtb* dose. At 4- and 12-weeks post-infection, groups of mice (n=4-5) were sacrificed; lungs and spleen were collected for bacterial quantification. Tissues were homogenized (BioGen Pro200) in 3 ml of PBS containing 0.05% Tween-80 (PBS-T), serially diluted in PBS-T and plated on Middlebrook 7H10 agar (Difco) supplemented with 0.5% glycerol, 10% oleic acid-albumin-dextrose-catalase (OADC, Difco) and 100 μg/ml cycloheximide. Plates were incubated for 3-4 weeks at 37˚C before counting CFU.

## 3 Results

### 3.1 Generation of rP18tri viral vector vaccines encoding *Mtb* antigens

The genome of wild-type PICV consists of two RNA segments, each encoding two genes in opposite orientations (25). The L segment encodes a matrix protein Z and a large RNA polymerase L, while the S segment encodes the envelope glycoprotein GPC and the nucleoprotein NP (28). By splitting the S segment into the S1 and S2 segments, we were able to insert an ORF of up to 2 kilobases in each segment (**Figure 1A**) (21, 22).

We focused on two immunodominant *Mtb* antigens EsxH/TB10.4 and Ag85B as they have well-established major histocompatibility complex (MHC) class I (MHC-I) and MHC-II tetramer assays to conveniently evaluate the antigen-specific CD8 and CD4 T cells, respectively, in mice (29–33). To evaluate whether multiple antigens expressed from the same vector would interfere with the immune development, we included additional antigens ESAT-6/EsxA, Rv1733c, and RpfA that are associated with different stages of *Mtb* infection (acute, latency, and resuscitation, respectively) and that have been included in other candidate TB vaccines (12). To maximize the coding capacity of an ORF, we used a P2A linker between two antigens. The P2A linker sequence induces ribosomal skipping during translation, allowing the production of multiple proteins from one ORF (26, 34). We aim to test whether effective immune responses are induced to each antigen linked by the P2A sequence. This may represent a valid strategy to generate rP18tri-based multivalent vaccines, which confer a greater protection against disease than those containing a single antigen (33, 35, 36).

Codon-optimized genes were cloned into either the S1 or S2 vector as illustrated in **Figure 1A**. Recombinant rP18tri-based TB vaccines, TBvac-1, TBvac-2, and TBvac-10, were generated using reverse genetics as described previously (21, 22, 37). TBvac-1 and TBvac-2 encode the same *Mtb* antigens, Ag85B and EsxA-EsxH, except that the antigen location is swapped between the S1 and S2 segments (**Figure 1A**). TBvac-10 encodes Ag85B-EsxH within the S1 ORF, and RpfA-Rv1733c within the S2 ORF (**Figure 1A**). In this study, we used TBvac-2 and TBvac-10 to characterize T cell immune responses and evaluated TBvac-1 and TBvac-2 for protective efficacy against virulent *Mtb* challenge.

To detect antigen expression from viral vectored vaccines, Vero cells were mock infected or infected with rP18tri vector or viral vectored TB vaccines at MOI of 0.1 for 24 h. Ag85B expression was detected in cells infected with vaccines but not with vector or mock infection by both Western blotting (**Figure 1B**) and immunofluorescence assay (IFA) (**Figure 1C**). We also detected EsxA expression in cells infected with TBvac-1 and TBvac-2 but not with mock, vector, or TBvac-10 by IFA (**Figure 1C**). We were unable to assess EsxH, RpfA or Rv1733c expression due to lack of antibody reagents.

### 3.2 TBvac-2 induces functional antigen-specific T cell responses by ELISpot assay

**Figure 2:**
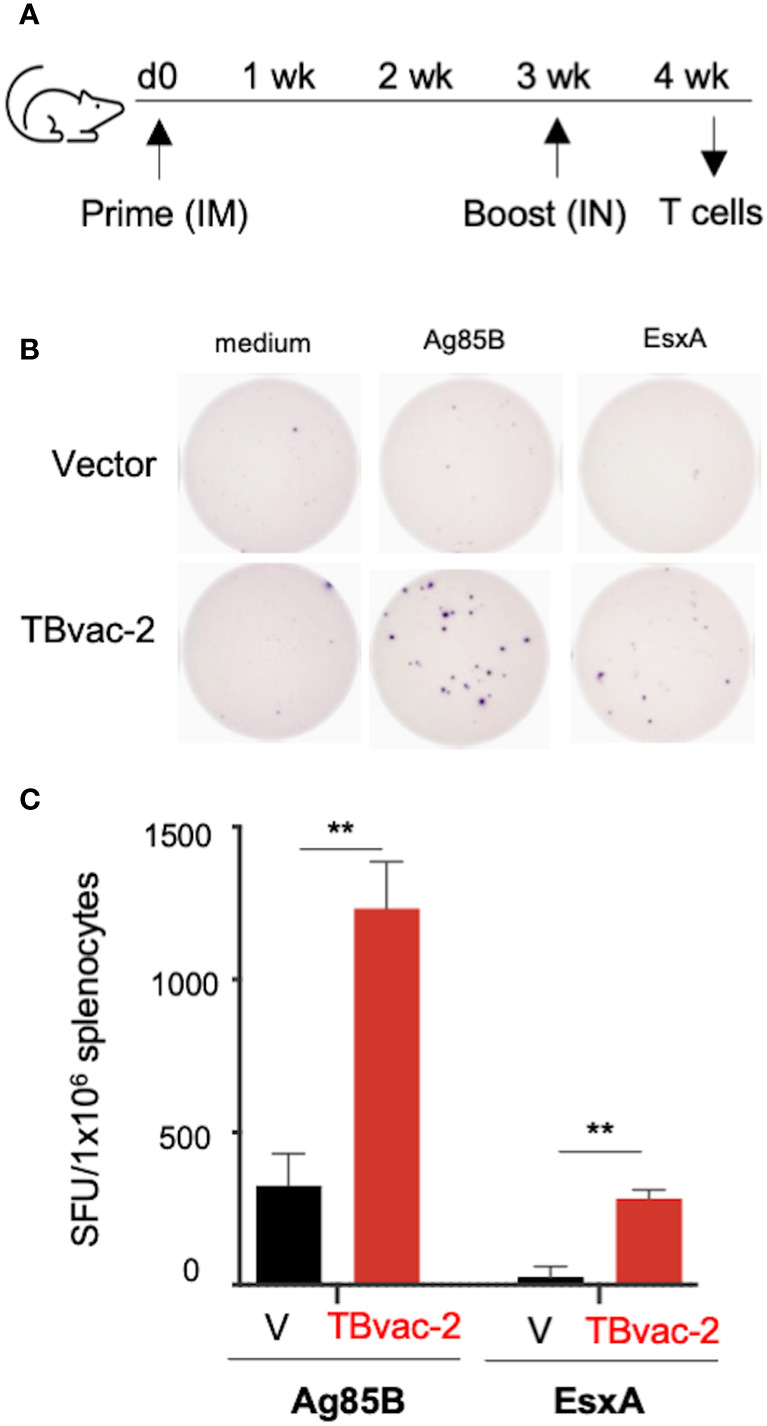
Evaluation of TBvac2-induced T cell responses by ELISpot assay. **(A)** BL6 mice (N=3) were primed with 1x10^5^ PFU of rP18tri viral vector (V) alone or TBvac-2 through the IM route, and 21 days later boosted with the same virus at the same dose through the IN route. At 7 d post-boost (dpb), splenocytes were isolated and incubated with medium alone or peptide pools of Ag85B or ESAT-6/EsxA, followed by detection of mouse IFNγ by ELISpot. **(B)** Representative ELISpot wells. **(C)** The number of IFNγ-secreting cells per million splenocytes in the vector and TBvac-2-immunized mice after Ag85B or ESAT-6/EsxA peptide stimulation was normalized with medium-alone control and shown as the average with standard deviation. Statistical analysis was conducted with unpaired t-test. **p < 0.01.

We first evaluated vaccine-induced T cell responses by IFNγ-based enzyme-linked immunosorbent spot (ELISpot) assay, which detects production of IFNγ from lymphocytes after antigen stimulation. We have previously shown that the rP18tri vector-based influenza vaccine induces even stronger antibody and T cell responses upon boost than after prime (21), so we evaluated T cell responses only after the boost dose. C57BL/6J (BL6) mice (n=3) were immunized with either the rP18tri vector (V) or TBvac-2 by an IM-IN prime-boost strategy (**Figure 2A**). At 7 days post-boost (dpb), splenocytes were analyzed by mouse IFNγ-based ELISpot assay after stimulation with medium alone or respective peptide pools (**Figure 2B**). After normalization to the medium control, the number of IFNγ-secreting cells after Ag85B or ESAT-6/EsxA peptide stimulation was significantly increased in TBvac-2 immunized compared to vector immunized mice (**Figure 2C**). These findings show that immunization with TBvac-2 elicits IFNγ-producing, antigen-specific T cell responses.

### 3.3 TBvac-2 and TBvac-10 induce strong antigen-specific CD8 T cells through both IM and IN routes

**Figure 3:**
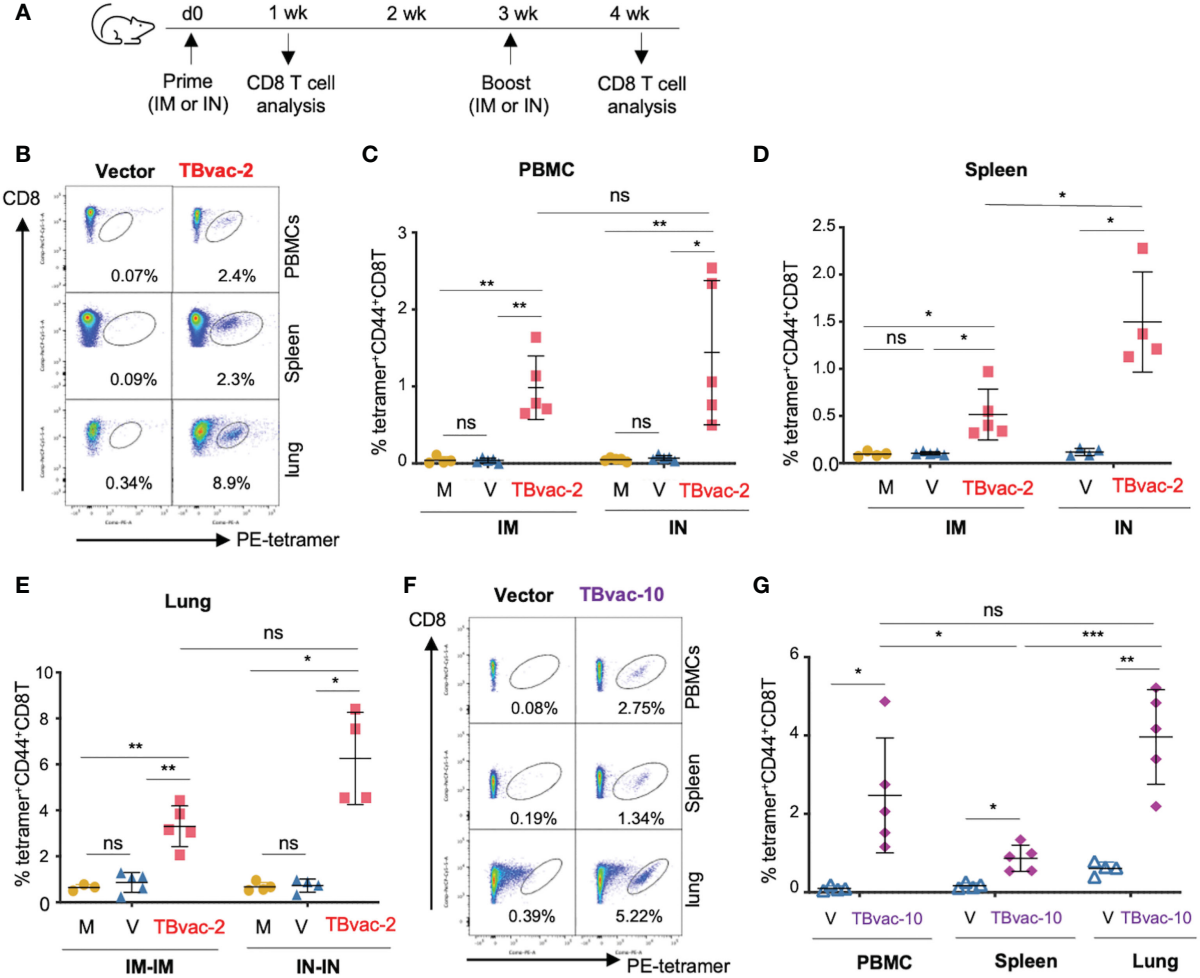
rP18tri-based TB vaccines induced strong antigen-specific CD8 T cells through both IM and IN routes. **(A)** BL6 mice (N = 4-5) were immunized with PBS (M), rP18tri vector alone (V), or TBvac-2 through either IM or IN route. Lymphocytes from peripheral blood (PBMC), spleen, and lung were incubated with PE-labeled MHC-I EsxH tetramer, along with antibodies against surface markers (CD3, CD8, and CD44), and analyzed by flow cytometry. Representative flow cytometry plots of the tetramer-positive CD44^h^CD8 T cells in peripheral blood (PBMC), spleens, and lungs from vector and TBvac-2-immunized mice are shown **(B).** The percentage of tetramer-positive CD44^h^CD8T cells in PBMCs **(C)** and spleens **(D)** is shown for each group (M, V, and TBvac-2) at 7 dpp through IM or IN route. The percentage of tetramer-positive CD44^h^CD8T cells in lungs **(E)** is shown for each group (M, V, and TBvac-2) at 7 days post-boost (dpb) through IM-IM or IN-IN route. A similar study was conducted to evaluate the TBvac-10-induced CD8T cells. BL6 mice (N=5) were immunized with rP18tri vector (V) or TBvac-10 through IM-IN prime-boost strategy. Representative flow cytometry plots **(F)** and the percentage **(G)** of tetramer-positive CD44^h^CD8T cells at 7 dpb in PBMCs, spleens, and lungs from vector and TBvac-10-immunized mice are shown. Statistical analysis was conducted with unpaired t-test. ***p < 0.001; **p < 0.01; *p < 0.05; ns, not significant.

We next evaluated the vaccine-induced antigen-specific CD8 T cell responses after either systemic (IM) or mucosal (IN) inoculation using an established MHC-I EsxH tetramer assay. BL6 mice were immunized with PBS/mock, rP18tri vector alone, or TBvac-2 through either IM or IN route (**Figure 3A**). At 7 d post-prime (dpp), lymphocytes from peripheral blood (PBMC), spleen, and lung were analyzed by EsxH-specific MHC-I tetramer analysis (**Figure 3B**). A single dose immunization of TBvac-2 through either IM or IN route generated a significantly higher percentage of MHC-I EsxH tetramer-positive CD8 T cells in the peripheral blood (**Figure 3C**) and spleen (**Figure 3D**) than mock or vector immunization. No significant difference in the PBMC CD8 T cell response was detected between the IM and IN routes (**Figure 3C**), while IN appears to elicit higher CD8 T cell responses than IM in the spleen (**Figure 3D**). We also quantified the tetramer-positive CD8 T cells in the lung at 7 d post-boost after prime-boost immunization by the IM-IM or IN-IN routes (**Figure 3A**). Consistent with the results observed in PBMCs and spleen, TBvac-2 immunization *via* both routes generated a significantly higher percentage of tetramer-positive CD8 T cells in the lung than mock or vector alone, with a trend toward higher responses in mice immunized by the IN route compared to IM, though this did not achieve statistical significance (**Figure 3E**).

As both routes of inoculation effectively stimulated immunity, we elected to move forward with a heterologous prime-boost strategy (IM-IN), which has been shown to result in greater protection against *Mtb* in guinea pigs (38). We evaluated CD8 T cell responses induced in mice immunized IM-IN with TBvac-10 by the MHC-I EsxH tetramer assay (**Figure 3F**). Mice immunized with TBvac-10 had a significantly higher percentage of tetramer-positive CD8 T cells compared to vector immunized animals in the peripheral blood, spleen, and lung (**Figure 3G**). The percentage of antigen-specific CD8 T cells was significantly higher in the lung than in the spleen.

Taken together, these results show that two different rP18tri-based TB vaccines (TBvac-2 and TBvac-10) administered systemically, mucosally, or a combination of the two, elicit strong antigen-specific CD8 T cells in the spleen, peripheral blood, and lungs. As EsxH is expressed after the P2A linker in both TBvac-2 and TBvac-10, these results also demonstrate that P2A-linked antigens in the rP18tri viral vector can stimulate robust immune responses.

### 3.4 TBvac-2 and TBvac-10 induce functional antigen-specific CD4 T cells in the lungs

**Figure 4:**
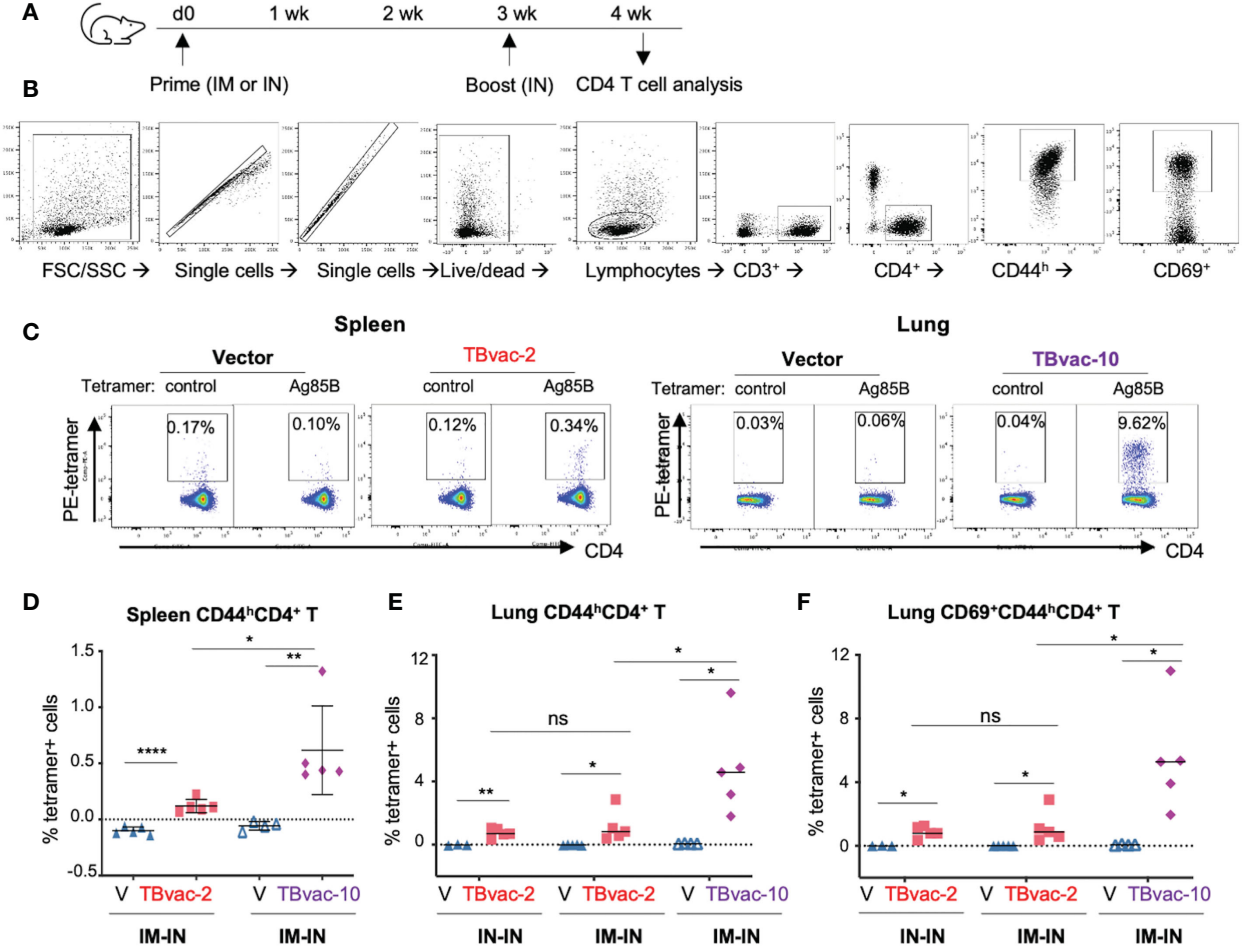
rP18tri-based TB vaccines induced strong antigen-specific CD4 T cells. **(A)** BL6 mice (N=5) were immunized with rP18tri vector (V), TBvac-2, and TBvac-10 through IM-IN or IN-IN prime-boost strategy. At 7 dpb, lymphocytes isolated from spleens or lungs were incubated with a PE-labeled control or Ag85B MHC-II tetramer, together with antibodies against cell surface markers (CD3, CD4, CD8, CD44, CD69). **(B)** Gating strategy used to analyze the MHC-II tetramer-positive CD44^high^ (CD44^h^) CD4^+^ T cells and lung CD69^+^CD44^h^CD4^+^ T cells. **(C)** Representative flow cytometry plots of control- and Ag85b-tetramer-positive CD44^h^CD4^+^ T cells were shown for splenocytes of vector and TBvac-2 immunization, and for lung lymphocytes in vector and TBvac-10 immunization. After normalization with the control MHC-II tetramer, the Ag85B tetramer-positive spleen CD44^h^CD4^+^ T cells **(D)**, lung CD44^h^CD4^+^ T cells **(E)**, and lung CD69^+^CD44^h^CD4^+^ T cells **(F)** was shown for vector (V), TBvac-2, and TBvac-10 immunization. Statistical analysis was conducted with unpaired t-test. ****p < 0.0001; **p < 0.01; *p < 0.05; ns, not significant.

We next evaluated the vaccine-induced CD4 T cell responses by MHC-II tetramer analysis. BL6 mice were prime-boosted with vector (V), TBvac-2, or TBvac-10 by either the IM-IN or IN-IN routes (**Figure 4A**). Spleen and lung lymphocytes were analyzed for Ag85B MHC-II tetramer-positive CD4 T cells (**Figure 4B**). A gating strategy for analyzing the flow cytometry data is shown in **Figure 4B**. Both spleen and lung lymphocytes were gated for CD3^+^CD4^+^CD44^h^ population and lung lymphocytes were further gated by CD69^+^, which is a tissue-resident cell surface marker (39). Tetramer-positive cells in the target cell populations were specifically detected in TBvac-2 and TBvac-10-immunized mice after incubation with Ag85B MHC-II tetramer (**Figure 4C**). After normalization with the control MHC-II tetramer, the percentage of Ag85B MHC-II tetramer-positive cells among CD4 T cells in the spleen (**Figure 4D**) and lung (**Figure 4E**) were determined for each mouse. In both tissues, significantly higher percentages of tetramer-positive cells were detected in mice immunized with TBvac-2 and TBvac-10 than with the vector, demonstrating the induction of antigen-specific CD4 T cells by both vaccines. TBvac-10 induced a higher percentage of tetramer-positive CD4 T cells in both the spleen (**Figure 4D**) and lung (**Figure 4E**) compared to TBvac-2, suggesting a stronger Ag85B-specific CD4 T cell immunity. No significant differences in lung tetramer-positive CD4 T cells were detected between IN-IN and IM-IN routes of inoculation with TBvac-2 (**Figure 4E**).

Recent studies suggest that tissue-resident memory T cells (Trm) play an important role in the primary defense against infection (40) and that *Mtb*-specific CD4 and CD8 T cells with tissue-resident memory phenotypes are associated with protection (12). To determine whether the vaccine-induced lung CD4 T cells exhibit tissue-resident phenotypes, we gated the CD69^+^ (a tissue-resident cell marker) population of the CD4 T cells (CD3^+^CD4^+^CD44^h^CD69^+^) for quantification of tetramer-positive cells. Both TBvac-2 and TBvac-10 stimulated high levels of tetramer-positive CD69^+^ CD4 T cells in the lung (**Figure 4F**). Consistent with the results for CD4 T cells (**Figures 4D, E**), TBvac-10 immunization induced a higher level of CD69^+^CD44^h^CD4 T cells than TBvac-2 (**Figure 4F**).

**Figure 5:**
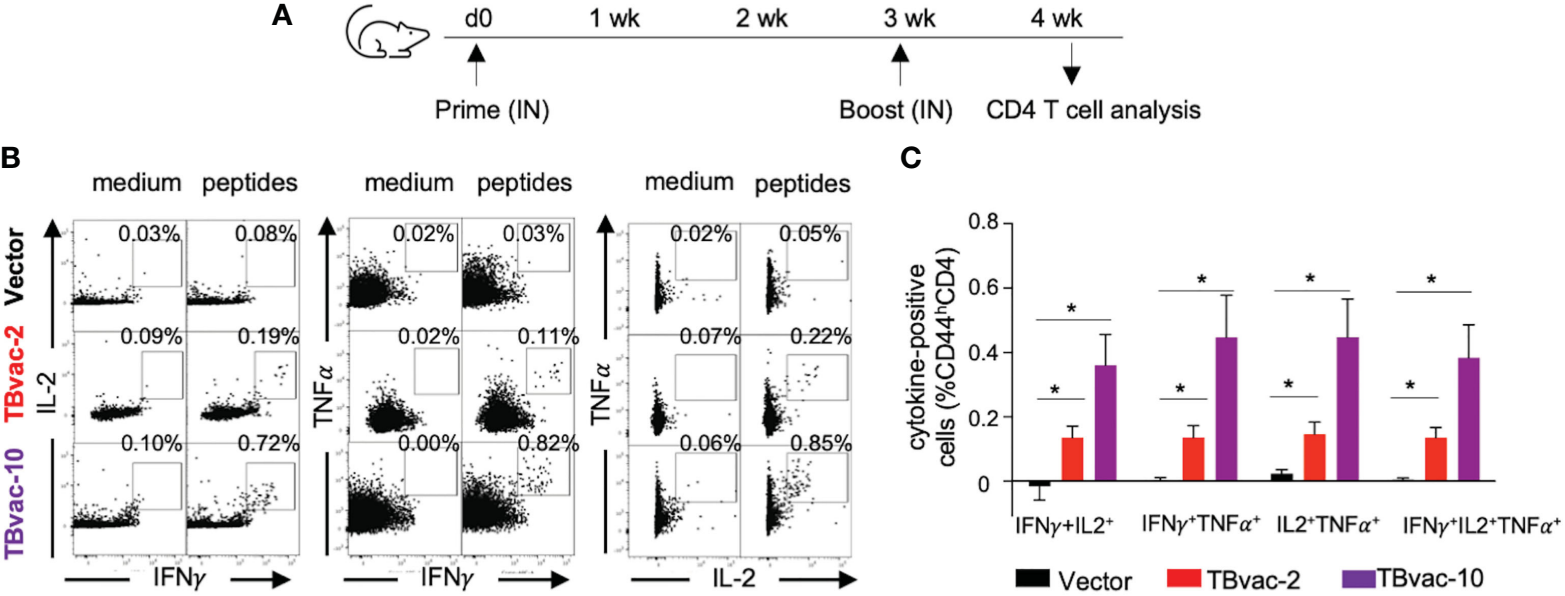
rP18tri-based TB vaccines induced multifunctional CD4 T cells. **(A)** BL6 mice (N=5) were immunized with rP18tri vector (V), TBvac-2, and TBvac-10 through IN-IN prime-boost strategy. At 7 dpb, PBMCs were collected and incubated in medium only or with pooled Ag85B/ESAT6 antigens, and stained for cell surface markers and cytokines. Representative flow cytometry plots of CD4 T cells (CD44^h^CD4^+^CD3^+^) positive for dual cytokine expression (IFNγ/IL-2, IFNγ/TNFα, and IL-2/TNFα) after either medium or peptide stimulation were shown for each immunization group **(B)**. After normalization with the medium control, the average percentages of dual- and triple-positive (IFNγ/IL-2/TNFα) CD4 T cells after peptide stimulation were shown for each group **(C)**. *p<0.05.

As multifunctional T cells are associated with control of multiple intracellular pathogens (41, 42) including *Mtb* (43), we also evaluated whether the viral vaccines can induce antigen-specific functional CD4 T cells by intracellular cytokine staining after *ex vivo* stimulation with antigen peptide pools (**Figure 5A**). CD4 T cells that were positive for dual cytokine expression (IFNγ/IL-2, IFNγ/TNFα, and IL-2/TNFα) were specifically detected after peptide stimulation in TBvac-2 and TBvac-10 immunized mice but not in vector-immunized mice (**Figure 5B**). The average percentages of dual- and triple-positive (IFNγ/IL-2/TNFα) CD4 T cells were significantly higher in mice receiving either vaccine than in the vector group (**Figure 5C**). There was a trend toward greater percentage of multifunctional cells for TBvac-10 than TBvac-2, but this was not statistically significant.

Taken together, these results suggest that both TBvac-2 and TBvac-10 can elicit functional antigen-specific CD4 T cells systemically and locally (lung) and that TBvac-10 may induce a stronger CD4 T cell immunity than TBvac-2.

### 3.5 rP18tri-based TB vaccines reduced bacterial load in mouse lungs in a virulent *Mtb* infection model

**Figure 6:**
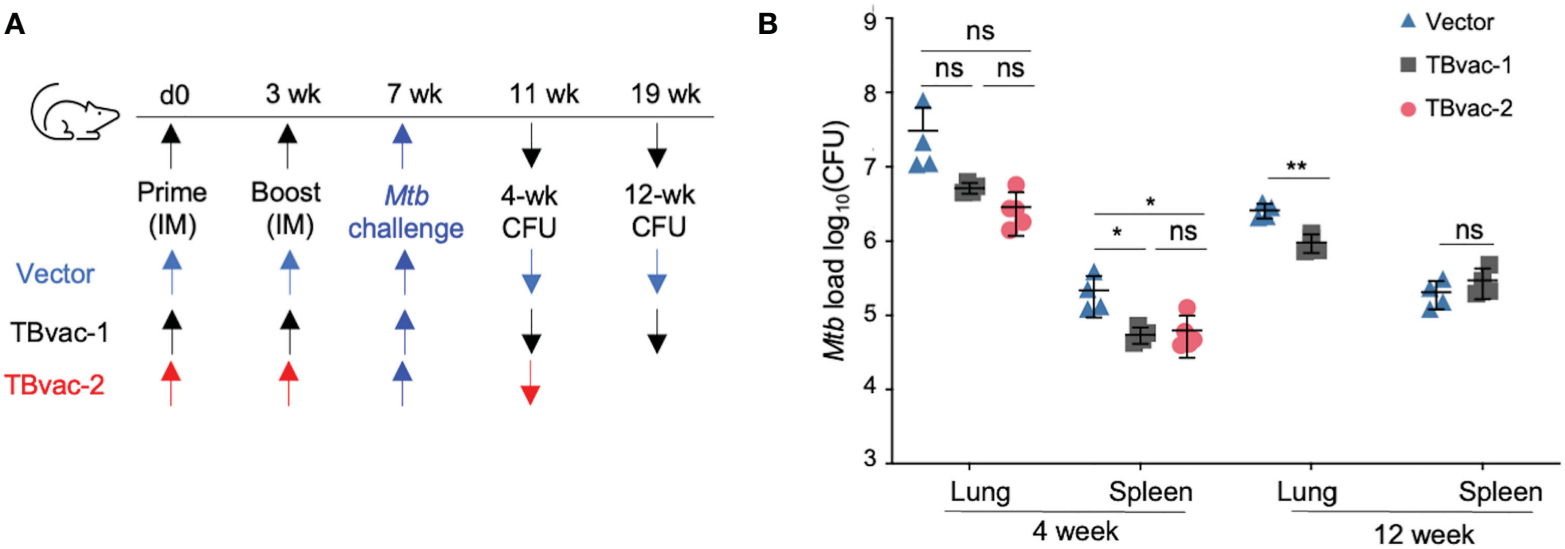
TBvac-1 and TBvac-2 reduced bacterial load in mouse lungs in a virulent *Mtb* infection model. **(A)** Illustration of the experimental procedure to evaluate the virus-induced protective efficacy in mice. Mice (n=4-5) were immunized (IM) through prime-boost strategy with vector or TBvac-1 or TBvac-2, and, 28 days later, challenged with virulent *Mtb*. **(B)** Tissues (lungs and spleens) were collected at 4 and 12 weeks after challenge to determine viable *Mtb* CFU by plating of tissue homogenates. Statistical analysis was conducted with unpaired t-test. **p < 0.01; *p < 0.05; ns, not significant.

We conducted an initial study to evaluate the protective efficacy of TBvac-1 and TBvac-2 in a standard *Mtb* low-dose aerosol challenge mouse model (44). BL6 mice prime-boost immunized through the IM route with 1 x10^5^ PFU of vector alone, TBvac-1, or TBvac-2 were infected by aerosol with virulent *Mtb* Erdman and euthanized at 4 or 12 weeks post-infection for bacterial load quantification (**Figure 6A**). At the 4-week timepoint, TBvac-1 and TBvac-2-immunized mice showed a similarly decreased bacterial load in both lungs and spleens (**Figure 6B**). The average *Mtb* burden in the lungs of TBvac-1 and TBvac-2-immunized mice decreased by 0.8 to 1 log compared to vector-immunized mice (**Figure 6B**). Although not statistically significant, this decrease is similar to what is seen in BL6 mice immunized with BCG alone (45, 46). TBvac-1 and TBvac-2 immunization also significantly lowered the bacterial load in the spleens at the 4-week time point compared to vector alone (**Figure 6B**). At the 12-week time point, we included the vector and TBvac-1 groups, but not TBvac-2 group, due to insufficient number of animals. TBvac-1 immunized animals had significantly decreased *Mtb* loads in the lung but not in the spleen (**Figure 6B**). These results establish that rP18tri-based vaccines expressing the *Mtb* antigens Ag85B, EsxA, and EsxH reduce the bacterial burden in mice challenged with virulent *Mtb*, demonstrating their potential for further development.

## 4 Discussion

An expanded pipeline of TB vaccine candidates is needed to ensure development of a highly effective TB vaccine. The goal of this study was to evaluate the immunogenicity and efficacy of novel multiantigen PICV-vectored TB vaccine candidates in mice. We show that immunization with these vaccine candidates elicits high levels of antigen-specific CD4 and CD8 T cells in the peripheral blood, spleen, and lung. These antigen-specific T cells are functional in secreting multiple cytokines (IFNγ, IL-2, and TNFα) upon antigen stimulation and express the CD69 tissue-resident marker in the lung. While CD4 T cells are traditionally thought to exert control over *Mtb* (8), recent work has shown that vaccine-induced CD8 T cells mediate protection in mice (47, 48). Thus, the PICV-vectored TB vaccine candidates have the potential to induce protective immunity by stimulating both arms of the T cell immune response. Indeed, we show that immunization with the PICV-vectored TB vaccines can reduce *Mtb* lung tissue burden and dissemination in an aerosol challenge mouse model. Future studies will evaluate the level of long-lived memory T cells and the duration of protection elicited by the live PICV vectored vaccines.

A major advantage of the PICV vector is its multiple delivery routes including systemic and mucosal sites that provide various options to efficiently control different pathogens. TB is a respiratory disease. Though IM vaccination is the most common route of immunization in people, recent studies indicate that mucosal immunization with TB vaccines results in robust local immunity (19, 20) and may be more protective (48, 49). We compared systemic (IM) and mucosal (IN) routes of the PICV-vectored vaccines in this study. Consistent with our previous findings (21), both routes of inoculation can elicit strong antigen-specific CD8 T cells after a single dose. The IN route seemed to elicit a higher CD8 T cell response than the IM route, both locally in the lung and systemically in spleen and blood, though the difference was not always statistically significant. We further showed that prime-boost *via* the IM-IN routes induced a similarly high level of CD4 T cells in the lung as *via* the IN-IN routes, suggesting that a mucosal boost immunization of the PICV-vectored vaccine is sufficient to induce effective lung T cell immunity. Our results are consistent with a published study showing that a systemic IM prime and IN boost of a viral vector vaccine elicited efficient T cell homing to the lungs (50).

Mucosal immunization with the live PICV vector is expected to elicit Trm cells. Upon IN inoculation, the PICV-vectored vaccines preferentially targets antigen-presenting cells (APCs) (13), which migrate to the mediastinal lymph node, where effector memory T cells can be activated, migrate into the lung tissue, and convert into Trm (51). Trm cells are located at barrier epithelial sites and rapidly respond to pathogens *via* cytotoxicity and cytokine production (39, 52). A protective role for Trm during *Mtb* infection has been shown in NHPs (49) and mice (53). Trm-mediated protection against *Mtb* in mice occurs following immunization with either mucosal BCG (48) or a recombinant influenza A virus-vectored vaccine (54). Functional Trm have also been identified in the respiratory tracts of people with active TB disease (55). The important role of Trm in *Mtb* control supports the development of mucosal TB vaccines designed to elicit Trm (56). Multiple viral vectored mucosal TB vaccines have been shown to effectively stimulate CD4 and/or CD8 Trm after respiratory immunization (19, 47, 54). We show here that the PICV-vectored vaccine candidates after the IN inoculation can elicit a high level of lung CD4 T cells expressing tissue-resident marker CD69. Whether these are Trm cells remains to be validated in future studies using intravascular staining and expanded Trm cell markers such as CD103 and CD11a (57, 58).

The three PICV-vectored vaccine candidates used in this study encode two common antigens EsxH and Ag85B. TBvac-10 elicits more Ag85B-specific circulating and lung tissue-resident CD4 T cells than TBvac-2, possibly due to an increased antigen production and/or presentation. Ag85B is expressed alone for TBvac-2, but linked to EsxH through a P2A linker for TBvac-10. These differences may affect when, where, and how much Ag85B is expressed in viral vaccine-infected cells, leading to differential magnitude of T cell responses. Nevertheless, studies with TBvac-10 suggest that antigens linked by P2A sequences can each induce strong T cell responses. Thus, inclusion of multiple antigens utilizing the two ORFs within the PICV vector is feasible, and is an important strategy for developing future TB vaccine candidates, as other vaccines incorporating multiple antigens show improved efficacy against *Mtb* (35, 36). The *Mtb* antigens tested in this study represent a small subset of T cell targets. They were selected for convenient quantification of T cell responses but these antigens such as EsxA and Ag85B are suboptimal for inducing protective immunity (32). Incorporation of new immunogens in the PICV vector platform, using the strategy validated in this study, has the potential to increase protection.

We demonstrated that systemic (IM) immunization of PICV-based TB vaccine candidates could partially protect mice from virulent *Mtb* infection in an aerosol infection model. Two candidates (TBvac-1 and TBvac-2) expressing the same three *Mtb* antigens (Ag85B, EsxH, and ESAT-6/EsxA), decreased bacterial load in the lung and the spleen by ~ 1 log at the 4-week time point in *Mtb*-infected mice compared to the viral vector alone. TBvac-1 (TBvac-2 not tested) also reduced the bacterial load in the lung but not in the spleen at the 12-week time point. This might be explained by the delayed *Mtb* dissemination from the lung to the spleen, or less durable protection in the spleen than in the lung. Future studies to analyze the long-lived memory cells at both lung and spleen infection sites will help address this question. A relatively high dose (~400 CFU) of virulent *Mtb* was used in the challenge model, which might have obscured some protective effect of the vaccines. As a mock-immunized group was not included in the protection experiment, we could not determine whether general immune stimulation induced by the PICV vector could further contribute to protection. Such vector-induced protection has been documented with other viral vectors (59) and will be important to evaluate in further studies. Nevertheless, the antigen-specific immunity elicited by TBvac-1 and TBvac-2 was able to decrease the bacterial load by ~ 1 log compared to the vector group, similar to the level of protection induced by BCG vaccination in the low-dose aerosol mouse model (45, 46). The three TBvac candidates in this study express the few well-characterized antigens, mainly for the purpose of T cell analysis. Future studies will generate new PICV-vectored vaccines incorporating additional TB antigens and fully evaluate the vaccine-induced protective efficacy *via* the IN route either acting alone or as boosters for BCG (47).

In summary, we have shown that novel PICV-based TB vaccines elicit strong systemic and lung T cell immunity, and confer protection against *Mtb* infection in a virulent mouse model. Our study supports the continued development of multiantigen TB vaccine candidates using the PICV vector platform.

## Data availability statement

The original contributions presented in the study are included in the article/supplementary material. Further inquiries can be directed to the corresponding authors.

## Ethics statement

The animal study was reviewed and approved by the Institutional Animal Care and Use Committee (IACUC) of University of Minnesota, Twin Cities.

## Author contributions

HL, AT, and YL contributed to conception and design of the study. NK, SV, QH, MR, AB, DW, SN, and HM generated data. NK, AT, and YL contributed to data analysis. NK and YL performed statistical analysis. NK and YL drafted the manuscript. AT and YL revised the manuscript. HL and MR contributed to manuscript edition. All authors contributed to the article and approved the submitted version.
